# Effect of carbon black nanomaterial on biological membranes revealed by shape of human erythrocytes, platelets and phospholipid vesicles

**DOI:** 10.1186/s12951-015-0087-3

**Published:** 2015-03-28

**Authors:** Manca Pajnič, Barbara Drašler, Vid Šuštar, Judita Lea Krek, Roman Štukelj, Metka Šimundić, Veno Kononenko, Darko Makovec, Henry Hägerstrand, Damjana Drobne, Veronika Kralj-Iglič

**Affiliations:** Laboratory of Clinical Biophysics, University of Ljubljana, Faculty of Health Sciences, Zdravstvena pot 5, Ljubljana, SI-1000 Slovenia; Group of Nanobiology and Nanotoxicology, University of Ljubljana, Biotechnical Faculty, Večna pot 111, Ljubljana, SI-1000 Slovenia; Lymphocyte Cytoskeleton Group, Institute of Biomedicine/Pathology, BioCity, University of Turku, Tykistökatu 6B, Turku, SF-20520 Finland; J. Stefan Institute, Jamova 39, Ljubljana, SI-1000 Slovenia; Department of Biosciences, BioCity, Åbo Akademi University, BioCity, Artillerigatan 6, Åbo/Turku, SF-20520 Finland

**Keywords:** Carbon black nanomaterial, Carbon black, Air pollution, Nanotoxicity, Cell membranes, Phospholipid bilayer, Model systems, Blood cells

## Abstract

**Background:**

We studied the effect of carbon black (CB) agglomerated nanomaterial on biological membranes as revealed by shapes of human erythrocytes, platelets and giant phospholipid vesicles. Diluted human blood was incubated with CB nanomaterial and observed by different microscopic techniques. Giant unilamellar phospholipid vesicles (GUVs) created by electroformation were incubated with CB nanomaterial and observed by optical microscopy. Populations of erythrocytes and GUVs were analyzed: the effect of CB nanomaterial was assessed by the average number and distribution of erythrocyte shape types (discocytes, echinocytes, stomatocytes) and of vesicles in test suspensions, with respect to control suspensions. Ensembles of representative images were created and analyzed using computer aided image processing and statistical methods. In a population study, blood of 14 healthy human donors was incubated with CB nanomaterial. Blood cell parameters (concentration of different cell types, their volumes and distributions) were assessed.

**Results:**

We found that CB nanomaterial formed micrometer-sized agglomerates in citrated and phosphate buffered saline, in diluted blood and in blood plasma. These agglomerates interacted with erythrocyte membranes but did not affect erythrocyte shape locally or globally. CB nanomaterial agglomerates were found to mediate attractive interaction between blood cells and to present seeds for formation of agglomerate - blood cells complexes. Distortion of disc shape of resting platelets due to incubation with CB nanomaterial was not observed. CB nanomaterial induced bursting of GUVs while the shape of the remaining vesicles was on the average more elongated than in control suspension, indicating indirect osmotic effects of CB nanomaterial.

**Conclusions:**

CB nanomaterial interacts with membranes of blood cells but does not have a direct effect on local or global membrane shape in physiological *in vitro* conditions. Blood cells and GUVs are convenient and ethically acceptable methods for the study of effects of various substances on biological membranes and therefrom derived effects on organisms.

## Background

Recent advances in development and industrial production of nanomaterials require assessment of their effects on human and animal health. Toxicity of nanomaterials has therefore become an issue regarding the question which methods are the most appropriate for determining health risk [1,2]. Nanomaterial introduces also effects that cannot be explained by composition of the material and chemical reactions, but require consideration of non-specific properties, such as size and shape of particles, their electromagnetic properties and interactions with biological material [3]. Since the underlying mechanisms are largely unknown, standard methods for research, testing and safety are not necessarily relevant for these materials. Standard methods for research and testing of various compounds include experiments on experimental animals. These methods were found to have poor predictability regarding other species, in particular human, as shown by carcinogeneity studies [4-6]. It was suggested that *in vitro* research may provide essential information pertaining to the human health risks posed by nanoparticle exposure [7]. With fast and extensive development of new nanomaterials and their use in medicine and industry there is urgent need to develop effective and low cost methods that will enable understanding basic processes in living organisms. The role of nonspecific biophysical mechanisms has hitherto been underestimated as potentially essential in revealing biological processes but should be considered in future paradigms of research and testing of nanomaterials.

Exogenously added substances first come in contact with cells by interacting with the membrane, so it is of great interest to study the interaction of these substances with biological membranes. Convenient systems for such studies are mammalian erythrocytes and giant unilamelar phospholipid vesicles (GUVs) composed of a closed bilayer membrane. These entities do not have internal structure (other than cortical membrane skeleton in case of erythrocytes) so their equilibrium shape is determined by the minimum of the membrane free energy [8]. Interaction of the added substance with the membrane causes changes in the membrane properties and in the constraints imposed upon the cell/vesicle (fixed area of the membrane, fixed difference between the areas of the two membrane layers, fixed cell/vesicle volume) which is reflected in the shape change [9]. Since mammalian erythrocytes and phospholipid vesicles (sized up to 100 micrometers) can be observed under the optical microscope, the effect of the exogenously added substances can be directly followed in real time.

Further, of special interest are processes caused by exogenously added substances that increase the risk for thromboembolic events [10]. Some parameters of these processes, e.g. activation of platelets and coalescence of membranous structures due to the presence of nanomaterial have previously been studied [11].

Membrane – nanomaterial interactions could be considered as one of the basic elements in an effective strategy of research and testing. These interactions are subject to biophysical methods which provide theoretical background. The accompanying experiments on the microscopic level are performed *in vitro*, on the artificial membranes and cell membranes.

Carbon black (CB) is a material produced by the incomplete combustion of carbon. Commercially available CB is a well characterized carbonaceous core particle that has been used extensively as a model for Diesel exhaust particles, which showed proinflamatory and prothrombotic systemic effects [12]. CB nanoparticles that are produced in traffic are present in the air, so living beings are commonly exposed to them. CB nanoparticles thus enter the body by means of inhalation. As they are very small, they cross biological barriers, enter the circulation and are spread in tissues and fetal organs. In the body, they affect the cell function and due to intercellular interactions [13] affect the entire organism. CB nanomaterial was found to have a size-dependent effect on *in vitro* cultures [14]. The reported effects of CB on animals include changes in development, in the immune response, and in gene expression [15]. Epidemiological studies on human have shown that CB nanomaterial is connected with adverse effects on health [16], in particular, with respiratory diseases and lung cancer.

In this work we studied the effect of CB nanomaterial on biological membranes as revealed by shape changes of erythrocytes and GUVs. These model systems and their interactions with the added substances have previously been used in the study of the effects of various compounds [17-21] including nanoparticles [11,22-26] on cells and their membranes and were found to contribute to better understanding of the relevant mechanisms which are common in *ex vivo* and *in vivo* exposures.

## Results

### Characterization and imaging of carbon black nanomaterial suspensions

**Figure 1:**
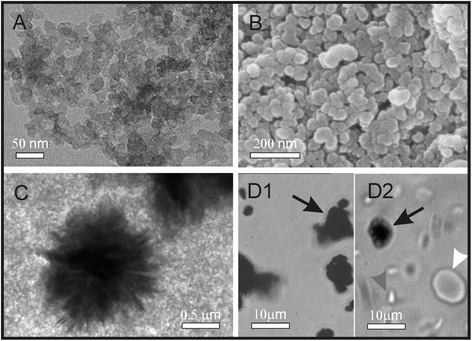
**Micrographs of agglomerated carbon black nanomaterial suspended in citrated and phosphate–buffered saline.** Agglomerated carbon black (CB) nanomaterial suspended in citrated and phosphate buffered saline (PBS) as observed by transmission electron microscope (TEM) **(A)** and scanning electron microscope (SEM) **(B)**. The primary particles are amorphous with homogeneous size about 20 nm. A small number of larger agglomerates were found in PBS, as observed by TEM **(C)** and by the optical microscope **(D1)**, and in platelet rich plasma **(D2)**. Black arrows point to agglomerates, white arrow points to an erythrocyte and gray arrow points to a platelet.

Transmission and scanning electron microscope (TEM and SEM, respectively) images of CB nanomaterial suspended in citrated and phosphate-buffered saline (PBS) showed agglomerates of globular nanoparticles of homogeneous size about 20 nm (Figure 1A and B, respectively) and a small amount of larger aggregates (Figure 1C). By using the optical microscope, we could inspect relatively large volume of the test sample. We observed even larger aggregates in PBS (Figure 1D1) and in platelet rich plasma (Figure 1D2). These large aggregates had different sizes, some of them extending over 10 μm (Figure 1D). Dynamic light scattering (DLS) measurements of previously sonicated CB nanomaterial showed a wide distribution of the agglomerates with respect to size (ranging from 500 to 2500 nm). Zeta potential of the PBS suspension of CB nanomaterial at pH = 7.32 was -29 mV. We could not perform DLS measurements of CB nanomaterial suspensions in 0.3 M glucose solution due to strong agglomeration and fast sedimentation of CB nanomaterial.

### Carbon black nanomaterial interactions and effects on erythrocyte shape

**Figure 2:**
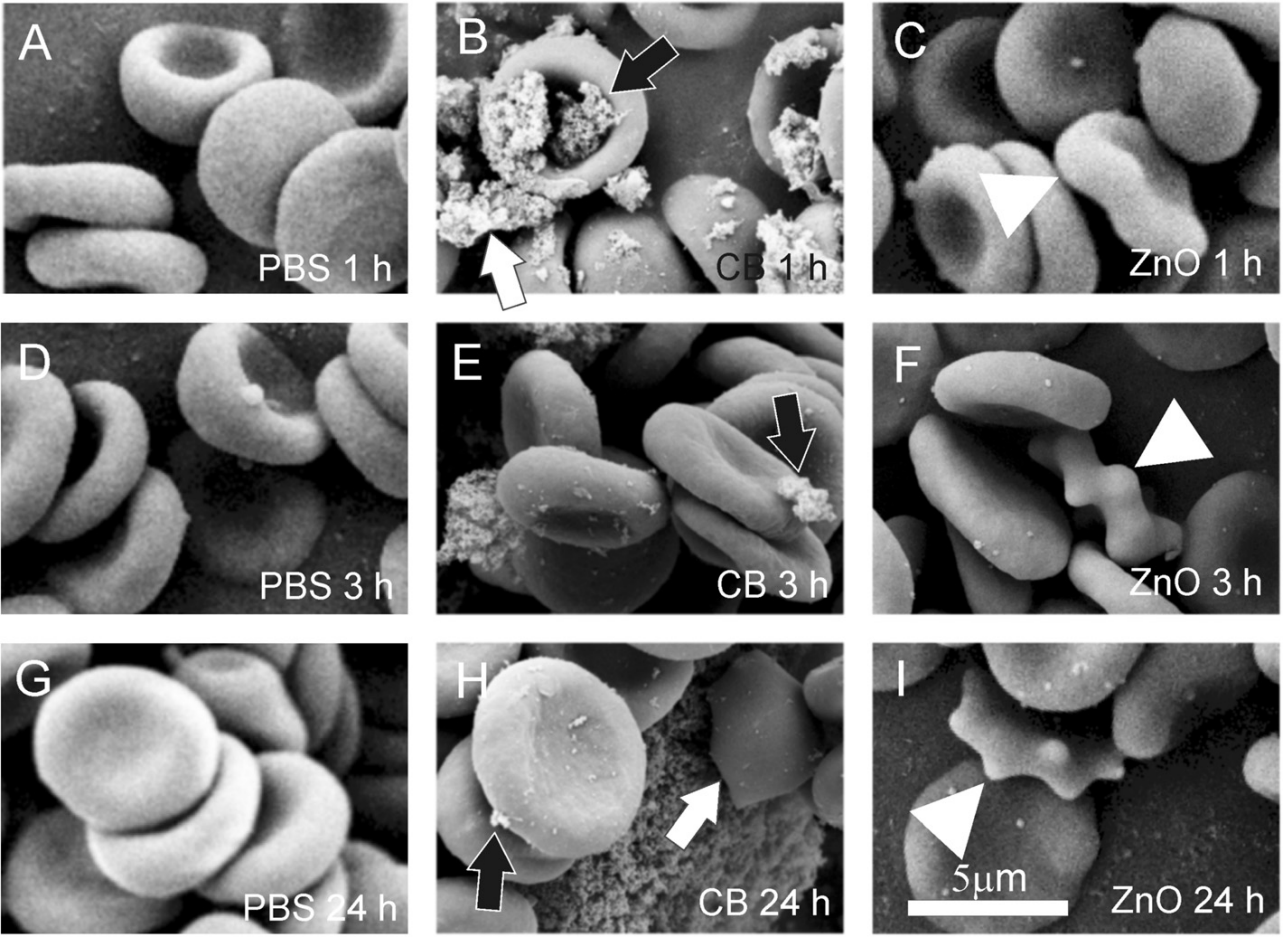
**The effect of carbon black nanomaterial on washed human erythrocytes.** The effect of carbon black (CB) nanomaterial on washed human erythrocytes as observed by scanning electron microscope. Panels **A**, **D** and **G** show the control samples with added citrated and phosphate buffered saline (PBS); panels **B**, **E** and **H** show the samples with added PBS-suspended CB nanomaterial after 1 hour, 3 hours and 24 hours of incubation, respectively and panels **C**, **F** and **I** show the samples with added PBS-suspended ZnO after 1 hour, 3 hours and 24 hours, respectively. Large CB agglomerates adhered to the erythrocyte surface (**B, E, H**, black arrows). Agglomerate which adhered to two erythrocytes mediated a bridging interaction between them (**B**, white arrow). In ZnO-treated samples, singular echinocytes were found (**C, F, I**, white triangles).

**Figure 3:**
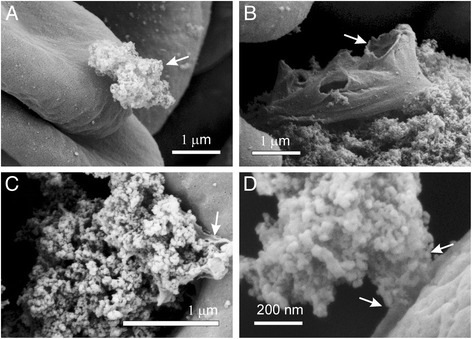
**Adhesion of large agglomerates of carbon black nanomaterial to the erythrocyte membrane.** In the case of a rather small contact area (panels **A, C** and **D**, white arrows) the local and the global membrane shape remained undisturbed. In the case of a large contact area **(B)** the membrane was considerably affected leading to formation of a ghost with large pores in the membrane (**B**, white arrow).

Figure 2 shows the effect of CB nanomaterial on the shape of washed human erythrocytes as observed by SEM. Panels A, D and G show the control samples (with added PBS) and panels B, E and H show the samples with added PBS-suspended CB nanomaterial after 1, 3 and 24 hours of incubation, respectively. For comparison, panels C, F and I show another control with added PBS-suspended zinc oxide nanoparticles (ZnO) [11]. Normal discocytic shapes of erythrocytes were observed after 1, 3 and 24 hours in all samples. Singular echinocytes were found in a positive control (ZnO-treated) samples ([11], Figure 2C, F and I). It can be seen in panels B, E and H that agglomerated CB nanomaterial adhered to the erythrocyte membrane. Large CB nanomaterial agglomerates have filled the discocyte dimple (Figure 2B, black arrow) but also agglomerates adhered to the membrane at any location on the erythrocyte surface (Figure 2E and H, black arrows). Large CB nanomaterial agglomerates can adhere to two cells and therefore mediate the interaction between them (Figure 2B, white arrow). A close-up view shows that in the case of small contact area between CB agglomerate and membrane (Figure 3A, C and D, white arrows), the CB nanomaterial does not affect the local membrane curvature (the membrane shape is undisturbed at the site of contact of the CB nanomaterial agglomerate). Moreover, the global shape of the cell remains discocytic (Figure 3A). However, singular disintegrated cells were found in the sample (Figure 3B). The cell shown in Figure 3B adhered to huge CB nanomaterial agglomerate and the shape was distorted. Large pores in the membrane (Figure 3B, white arrow) caused the exchange of the inner and the outer cell solutions. A similar process of cell deformation upon the adhesion to a huge agglomerate is indicated in Figure 2H (white arrow), but in this case, the membrane integrity is still preserved.

### Carbon black effects on erythrocyte shape in population of erythrocytes and platelets

**Figure 4:**
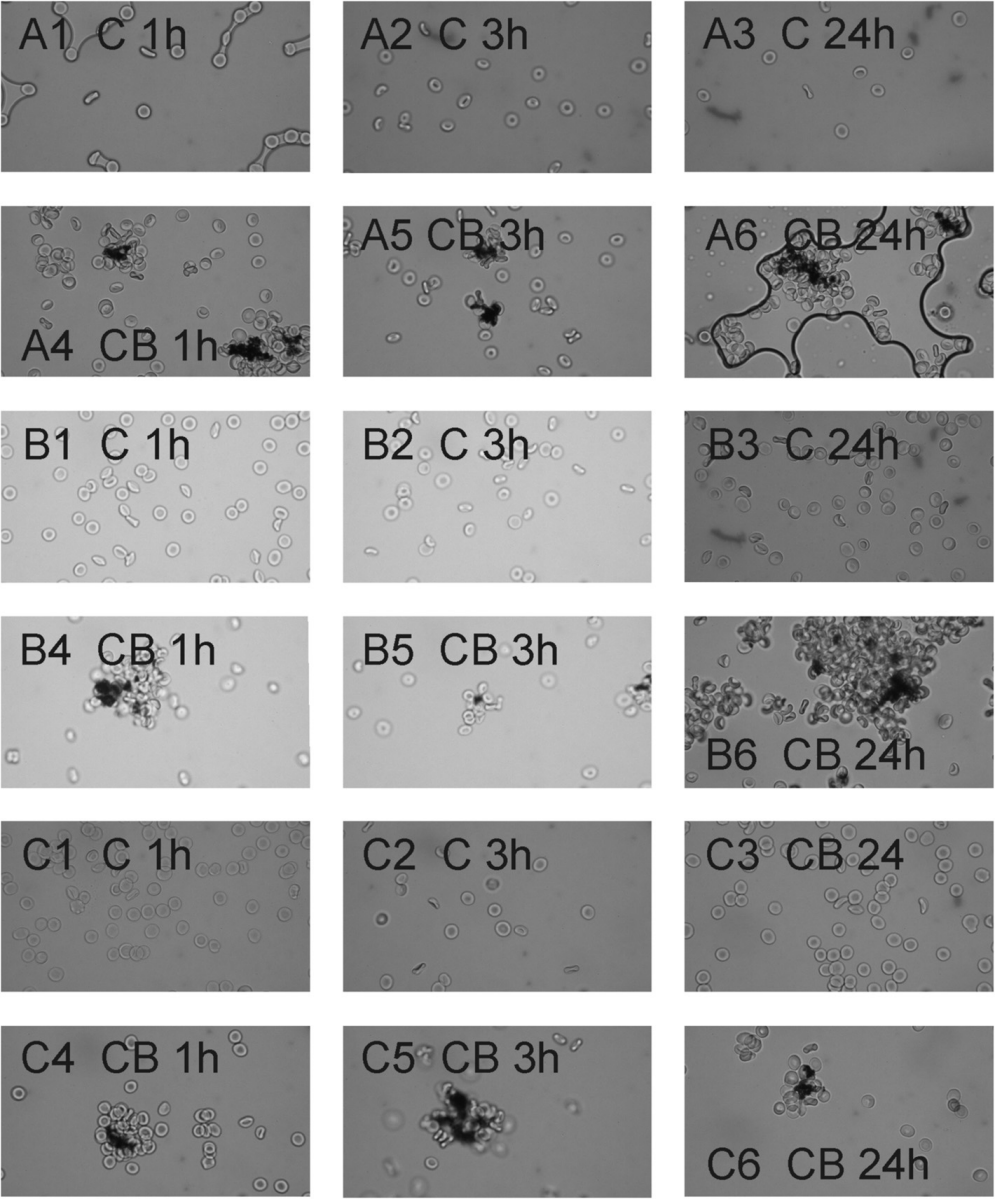
**The effect of carbon black nanomaterial on coalescence between erythrocytes.** The effect of carbon black (CB) nanomaterial on coalescence between erythrocytes in three subjects with no record of the disease (marked **A**, **B** and **C**) as observed by the phase contrast optical microscope. Washed erythrocytes were incubated with citrated and phosphate buffered saline (PBS) for control (denoted by **C**) and with PBS-suspended CB nanomaterial (denoted by **CB**) for 1, 3 and 24 hours, respectively, as indicated.

Figure 4 shows the effect of CB nanomaterial on coalescence of washed erythrocytes in three subjects with no record of the disease (A, B and C) as observed by the phase contrast optical microscope. Washed erythrocytes were incubated with PBS for control and with CB nanomaterial (suspended in PBS) for 1, 3 and 24 hours, respectively. It can be seen that the control samples remained unchanged after 1, 3 and 24 hours while in the test samples, the erythrocytes have gathered around larger CB nanomaterial agglomerates already after 1 hour. The size of the complexes remained essentially the same also after 3 and 24 hours (Figure 4). Erythrocytes that were not in direct contact with the visible CB nanomaterial agglomerates did not adhere to each other.

We analyzed the effect of CB nanomaterial on erythrocyte shape at the population level (Table 1). The examined blood initially contained mostly discocytes (Table 1). With time the portion of discocytes in the test sample and in the control sample decreased at the expense of the portion of echinocytes (Table 1), but the effect was stronger in the control sample. The differences between the respective test and control samples were statistically significant (p < 0.05) (Table 1). We observed no decreasing trend of the number of erythrocytes in the samples with time.

**Table 1:** **A population study of the effect of carbon black nanomaterial on erythrocyte shape**

	**Sample size (number of frames)**	**Test**			**Control**			**Difference between test and control**		
**Sample**	**Test/Control**	**D: % discocytes (SD)**	**E: % echinocytes (SD)**	**S: % stomatocytes (SD)**	**D: % discocytes (SD)**	**E: % echinocytes (SD)**	**S: % stomatocytes (SD)**	**ΔD (%) (p, P)**	**ΔE (%) (p, P)**	**ΔS (%) (p, P)**
1 hour	56/54	99 (2)	1 (1)	0	90 (8)	9 (7)	1 (5)	9 (<10^-6^, 1)	-8 (<10^-6^, 1)	NA
3 hours	50/53	90 (18)	9 (18)	0	84 (11)	16 (11)	0	6 (0.05, 0.62)	-6 (0.04, 0.75)	NA
24 hours	49/51	79 (9)	21 (9)	0	62 (9)	38 (10)	0	17 (<10^-6^, 1)	-17 (<10^-6^, 1)	NA

A population study of the effect of carbon black nanomaterial on erythrocyte shape. The average values of the portion of discocytes (D), echinocytes (E) and stomatocytes (S) are given after 1 hour, 3 hours and 24 hours of incubation with the carbon black nanomaterial (test sample) and with citrated and phosphate buffered saline (control sample). SD: standard deviation. p: statistical significance of the difference between the average values of the test and control, calculated by the *t*-test. P: statistical power. NA: non applicable as there were les than 1% of stomatocytes in all samples. Altogether 10712 cells were included in the analysis.

**Figure 5:**
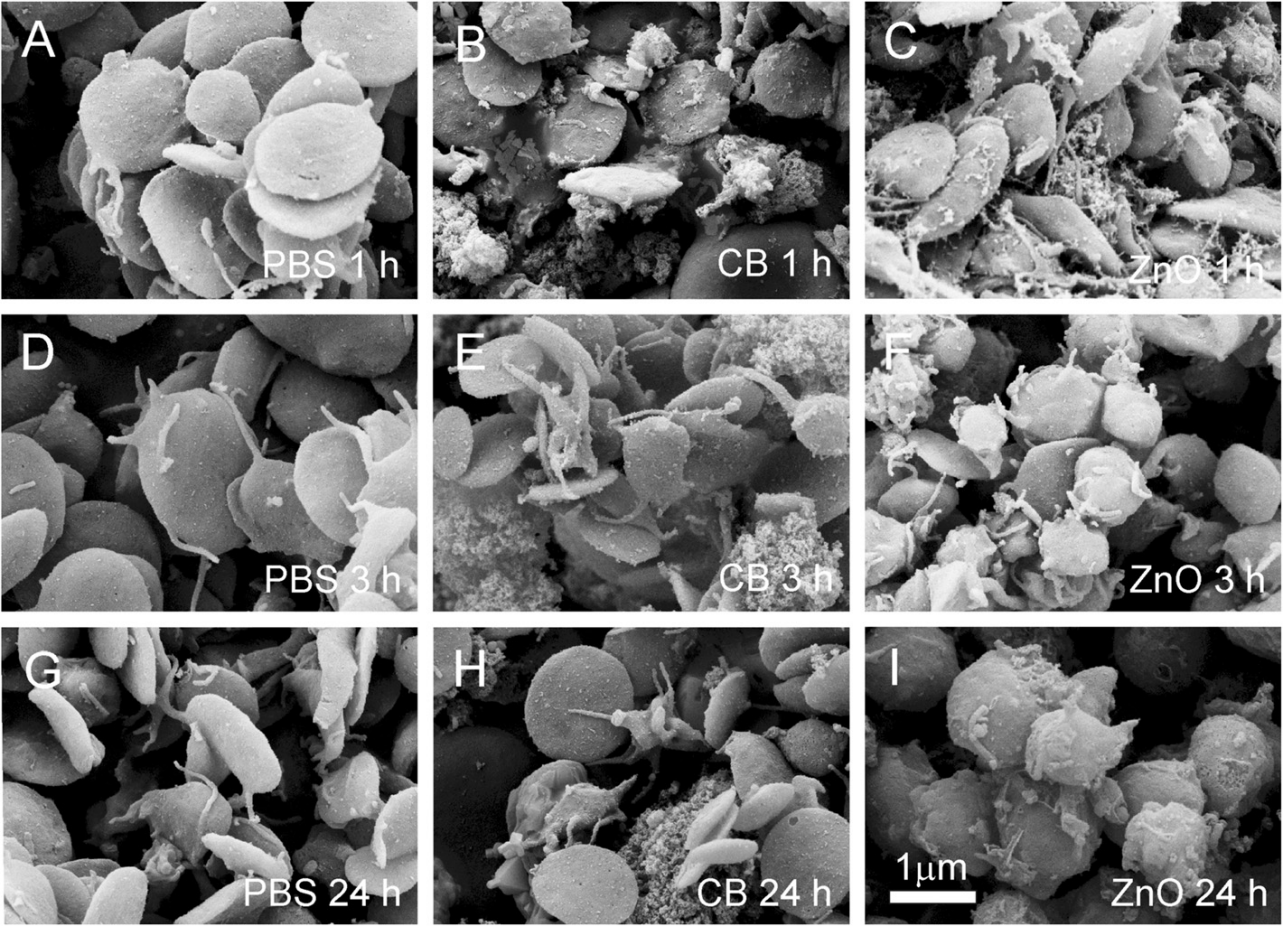
**The effect of carbon black nanomaterial on population of platelets.** The effect of carbon black (CB) nanomaterial on population of platelets was observed by the scanning electron microscope. Washed platelets were incubated with citrated and phosphate-buffered saline (PBS) for control **(A, D, G)**, with PBS-suspended CB nanomaterial **(B, E, H)** and for comparison, with ZnO [11] **(C, F, I)** for 1, 3 and 24 hours, respectively. After 1 and 3 hours the control and test samples contained disc-like platelets **(A, B, D, E)** characteristic for the resting state, while there were some platelets exhibiting deviation from the disc-like shape and tubular protrusions (characteristic for activated platelets) found after 24 hours **(E, F)**. For comparison, the effect of ZnO caused shape changes of some platelets after 3 hours **(F)** while after 24 hours all platelets attained swollen globular shape with remnants of tubular protrusions, indicating their activation **(G)**.

Figure 5 shows the effect of CB nanomaterial on population of platelets as observed by SEM. Washed platelets were incubated with PBS (control; A, D, G) and with CB nanomaterial suspended in PBS (B, E, H) for 1, 3 and 24 hours, respectively. For comparison, we show also platelets incubated with a positive control (ZnO agglomerated nanomaterial [11]) for 1, 3 and 24 hours, (C, F, I, respectively). After 1 and 3 hours, the control and test samples showed disc-like shapes (A, B, D, E) characteristic for resting platelets. There were some platelets exhibiting protrusions and shape deformation characteristic for activated platelets after 24 hours in the control and in the test sample (G,H). For comparison, the effect of ZnO nanoparticles caused shape transformation in some platelets after 3 hours (F) and swollen globular shapes with remnants of tubular protrusions characteristic for activated platelets after 24 hours (I).

### Carbon black effect on giant unilamellar phospholipid vesicles

**Figure 6:**
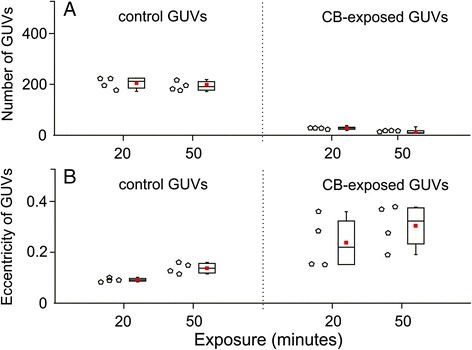
**The effect of carbon black nanomaterial on giant unilamellar vesicles.** Quantity of giant unilamellar vesicles (GUVs) incubated in chambers with 0.3 M glucose solution (control) and suspension of CB nanomaterial (0.05 mg/mL in 0.3 M glucose solution). Samples of GUVs were incubated with sugar solution for control and with carbon black (CB) nanomaterial suspended in sugar solution (0.05 mg/mL), for 20 minutes and 50 minutes, respectively. Each empty dot corresponds to a number of vesicles obtained in one sample, i.e. one replicate of each tested group. One sample of a population consists of all vesicles captured in two individual video tracks at different places in a chamber. Based on preliminary calculations, two such video tracks capture approximately 3% of the whole population of vesicles in the chamber. The red square in the center of the box plot represents the mean value of **(A)** the number of GUVs or **(B)** GUVs’ eccentricity, the central line is the median value, and the lower and upper lines of the box represent the 25th and 75th percentiles. The whiskers are set at two standard deviations from the mean value. Addition of CB nanomaterial considerably diminished the number of GUVs in the sample while the remaining GUVs exhibited an increased eccentricity of the contour (elongation).

Figure 6 shows the effect of CB nanomaterial on GUV abundance and shape. Incubation of GUVs with CB nanomaterial caused a considerable decrease in the number of GUVs already after 20 minutes (Figure 6A). The remaining GUVs in the test suspension exhibited higher eccentricity than the control GUVs (Figure 6B).

### Carbon black effect on parameters of blood cell populations

Tables 2, 3, 4 show blood cell population parameters (erythrocyte parameters: erythrocyte concentration RBC, hematocrit HCT, red cell distribution width RDW and hemoglobin distribution width HDW (Table 2), platelet parameters: concentration of platelets PLT, plateletcrit PCT, mean platelet volume MPV, platelet distribution width PDW, mean platelet complement MPC and mean platelet mass MPM (Table 3) and concentrations of leukocyte populations: total leukocytes (WBC), neutrophils, lymphocytes, monocytes, eosinophils and basophils, (Table 4) of samples incubated with two different concentrations of CB nanomaterial (0.5 mg/mL (A) and 1 mg/mL (B)), and control samples (incubated with equal volume of PBS). We found no differences between average values of parameters in test samples and in control samples with respect to number, mass and volume of erythrocytes and platelets and the shapes of their distributions (Tables 2 and 3). We found statistically significant difference in parameter MPC which measures refractive index of platelets (Table 3) and in the number of leukocytes; control samples had considerably less leukocytes than the control sample (20% (A) and 16% (B), respectively) (Table 4). Moreover, we found differences in distribution of events within the flow cytometer scatter diagram regions that are in blood samples attributed to different types of leukocytes (Table 4). The number of events within the eosinophils and basophils region was increased while the number of events within the monocyte region was decreased with respect to the control sample (Table 4). These differences were statistically significant and of sufficient statistical power (the power was considered sufficient if larger than 0.8). However, there were no differences between the respective samples incubated with different concentrations of nanomaterial (Table 4).

**Table 2:** **Erythrocyte parameters in blood samples incubated with carbon black nanomaterial**

**Parameter**	**Sample**	**1**	**2**	**3**	**4**	**5**	**6**	**7**	**8**	**9**	**10**	**11**	**12**	**13**	**14**	**AVG**	**STD**	**p**
RBC (10^12^ cells/L)	A	3.27	3.72	3.65	3.27	4.75	3.89	3.38	3.26	2.92	2.98	3.6	2.82	4.46	3.67	3.55	0.55	0.42 (A-C)
	B	3.51	3.56	3.5	3.41	4.46	3.84	3.39	3.44	3.35	2.5	3.95	3.23			3.51	0.46	0.27 (B-C)
	C	3.26	3.54	3.2	3.22	4.54	3.95	3.46	3.26	3.38	3.14	3.68	3.27	4.34	3.3	3.40	0.41	0.80 (A-B)
HCT (%)	A	0.28	0.32	0.30	0.28	0.40	0.34	0.31	0.28	0.26	0.26	0.32	0.24	0.37	0.31	0.31	0.04	0.96 (A-C)
	B	0.30	0.31	0.29	0.29	0.38	0.33	0.31	0.29	0.30	0.22	0.36	0.28			0.3	0.04	0.89 (B-C)
	C	0.28	0.31	0.27	0.27	0.39	0.34	0.32	0.27	0.30	0.27	0.33	0.28	0.36	0.28	0.3	0.04	0.76 (A-B)
RDW (%)	A	12.8	13.5	13	15.3	12.4	12.9	13.5	13.6	12.5	12.3	13	12.6	12.5	12.6	13.04	0.78	0.88 (A-C)
	B	12.7	13.5	13	15.4	12.4	12.8	13.4	13.6	12.8	12.4	12.9	12.6			13.13	0.82	0.90 (B-C)
	C	12.7	13.7	12.9	15.3	12.5	12.8	13.7	13.6	12.6	12.5	12.9	12.8	12.5	12.6	13.1	0.77	0.98 (A-B)
HDW (g/L)	A	24.5	22.5	27.1	26.3	24.6	24.0	22.6	25.7	27.2	24.1	23.4	22.7	25.9	25.9	24.8	1.62	0.95 (A-C)
	B	24.3	22.5	27.1	26.1	24.4	22.1	22.1	26.2	27.3	24.6	23.2	22.7			22.9	6.20	0.36 (B-C)
	C	24.6	22.7	26.8	26.2	24.7	23.9	22.3	26.2	27.5	24.3	23.5	22.6	25.9	25.9	24.8	1.66	0.37 (A-B)

Erythrocyte parameters - (Erythrocyte (RBC), Hematocrit (HCT), RBC distribution width (RDW) and hemoglobin distribution width (HDW)) in blood samples incubated with two different CB nanomaterial concentrations (A – 0.5 mg/mL, B - 1 mg/mL), and control suspension incubated with PBS (C). Average value (AVG), standard deviation (STD) and statistical significance of the difference between the test and the control samples (A-C and B-C, respectively) and between the two test samples (A-B), given by the respective probability (p).

**Table 3:** **Platelet parameters in blood samples incubated with carbon black nanomaterial**

**Parameter**	**Sample**	**1**	**2**	**3**	**4**	**5**	**6**	**7**	**8**	**9**	**10**	**11**	**12**	**13**	**14**	**AVG**	**STD**	**p**	**P**
PLT (10^9^ cells/L)	A	106	71	91	62	56	69	169	-	114	146	114	109	38	37	89.4	39.0	0.16 (A-C)	
	B	61	56	99	80	49	104	176	56	123	184	92	119			99.9	44.9	0.21 (B-C)	
	C	117	82	129	86	56	117	182	104	121	209	141	129	49	59	113	45.9	0.91 (A-B)	
PCT (%)	A	0.1	0.07	0.09	0.07	0.05	0.07	0.16	0.06	0.11	0.13	0.1	0.1	0.04	0.03	0.08	0.04	0.21 (A-C)	
	B	0.06	0.06	0.1	0.08	0.05	0.1	0.17	0.05	0.12	0.16	0.08	0.11			0.10	0.04	0.31 (B-C)	
	C	0.11	0.08	0.13	0.08	0.05	0.11	0.16	0.09	0.11	0.17	0.12	0.12	0.05	0.05	0.10	0.04	0.87 (A-B)	
MPV (fL)	A	9.5	10.5	9.9	10.5	9.2	9.7	9.4	9.2	9.6	8.6	9	8.9	9.4	8.3	9.41	0.63	0.62 (A-C)	
	B	10	10.5	10.2	9.9	9.3	9.6	9.6	8.8	9.6	8.4	8.7	9.4			9.5	0.63	0.39 (B-C)	
	C	9.5	10.3	10.1	9.7	9	9.5	8.8	8.7	9	8.4	8.8	9.6	9.7	9	9.29	0.56	1.00 (A-B)	
PDW (%)	A	56	63.8	57.6	65.4	72.2	61.3	62.1	67.7	59.1	54	61.9	53.8	51.6	47.7	59.6	6.67	0.15 (A-C)	
	B	61.5	61.0	59.7	56.8	63.2	61.8	61.8	54.5	56.8	55.4	47.2	60			58.3	4.49	0.31 (B-C)	
	C	54.6	62.3	55.4	54	60.2	58.1	58.3	59.7	52.9	52.4	60	50.9	56.3	57.8	56.6	3.39	0.17 (A-B)	
MPC (g/L)	A	215	211	216	209	223	222	228	209	215	219	219	229	206	174	214	13.4	0.02 (A-C)	0.88
	B	189	206	219	215	218	236	218	212	220	220	209	229			216	11.7	0.00 (B-C)	0.84
	C	214	225	222	232	226	239	232	233	230	230	229	229	219	193	225	11.2	0.61 (A-B)	
MPM (pg)	A	1.89	1.99	1.95	0.96	1.81	1.97	1.97	1.74	1.88	2.74	1.79	1.89	1.78	1.38	1.84	0.38	0.45 (A-C)	
	B	1.72	1.96	2.01	1.94	1.84	2.06	1.91	1.73	1.92	1.71	1.72	1.89			1.87	0.12	0.10 (B-C)	
	C	1.88	2.09	2.04	2.07	1.86	2.1	1.89	1.87	1.92	1.77	1.86	2.03	1.92	1.6	1.92	0.14	0.90 (A-B)	

Platelet parameters (Platelet count (PLT), plateletcrit (PCT), platelet distribution width (PDW), mean platelet volume (MPV), mean platelet mass (MPM) and mean platelet component (MPC)) in blood samples incubated with two different carbon black nanomaterial concentrations (A – 0.5 mg/mL, B – 1 mg/mL), and control suspension incubated with citrated and phosphate-buffered saline (C). Average value (AVG), standard deviation (STD) and statistical significance of the difference between the test and the control samples (A-C and B-C, respectively) and between the two test samples (A-B), given by the respective probability (p). Statistically significant differences have also been evaluated for statistical power at α = 0.05 (P).

**Table 4:** **Leukocyte parameters in blood samples incubated with carbon black nanomaterial**

**Parameter**	**Sample**	**1**	**2**	**3**	**4**	**5**	**6**	**7**	**8**	**9**	**10**	**11**	**12**	**13**	**14**	**AVG**	**STD**	**p**	**P**
WBC (10^9^ cells/L)	A	8.51	6.81	8.45	6.3	6.13	8.97	6.92	8.1	8.83	7.05	6.91	6.12	4.74	4.71	7.04	1.39	0.04 (A-B)	0.81
	B	9.04	5.97	8.12	5.78	6.33	8.28	7.12	7.29	8.94	8.55	8.3	7.46			7.60	1.13	0.00 (B-C)	1
	C	7.82	5.36	7.1	4.65	4.24	6.56	6.2	5.98	7.59	6.04	6.04	6.72	4.78	4.57	5.98	1.14	0.70 (A-B)	
Neutrophil region (10^9^ cells/L)	A	3.04	4.82	5.81	3.18	4.59	3.99	4.62	3.05	2.57	3.97	3.53	3.24	3.21	2.37	3.71	0.97	0.25 (A-B)	
	B	4.05	5.58	5.72	4.15	4.19	3.86	4.35	3.46	2.5	3.79	3.89	3.08			4.05	0.91	0.04 (B-C)	0.86
	C	3.79	4.12	5.29	2.93	3.74	3.68	3.72	2.29	1.98	3.33	3.22	2.89	3.05	2.29	3.31	0.86	0.37 (A-B)	
Lymphocyte region (10^9^ cells/L)	A	2.14	1.39	1.71	2.84	1.74	2.11	2.35	1.97	2.7	3.53	3.11	2.46	1.01	1.82	2.21	0.68	0.58 (A-B)	
	B	2.17	1.62	1.83	3.17	1.66	2.05	2.2	1.75	2.45	3.33	2.91	2.15			2.27	0.58	0.39 (B-C)	
	C	2.15	1.56	1.71	2.56	1.61	1.97	2.12	1.56	2.37	3.17	3.16	2.04	1.19	1.86	2.07	0.58	0.78 (A-B)	
Monocyte region (10^9^ cells/L)	A	0.14	0.19	0.23	0.27	0.22	0.23	0.24	0.24	0.08	0.2	0.18	0.15	0.25	0.15	0.20	0.05	0.00 (A-B)	1
	B	0.18	0.17	0.23	0.33	0.16	0.17	0.2	0.34	0.11	0.16	0.21	0.16			0.20	0.07	0.00 (B-C)	1
	C	0.4	0.24	0.36	0.43	0.31	0.39	0.36	0.3	0.22	0.3	0.36	0.3	0.34	0.18	0.32	0.07	0.87 (A-B)	
Eosinophil region (10^9^ cells/L)	A	0.69	0.38	0.95	0.76	1.41	0.44	1.61	0.72	0.8	0.63	1.6	0.9	0.2	0.3	0.81	0.45	0.00 (A-B)	1
	B	0.91	0.85	1.02	0.82	1.06	0.92	1.29	0.66	0.58	0.64	1.88	0.5			0.93	0.37	0.00 (B-C)	1
	C	0.35	0.08	0.19	0.09	0.3	0.12	0.31	0.07	0.06	0.27	1.04	0.09	0.17	0.19	0.24	0.25	0.49 (A-B)	
Basophil region (10^9^ cells/L)	A	0.1	0.14	0.14	0.28	0.13	0.14	0.14	0.15	0.15	0.1	0.1	0.06	0.06	0.07	0.13	0.06	0.00 (A-B)	1
	B	0.16	0.06	0.15	0.07	0.2	0.12	0.23	0.12	0.14	0.2	0.15	0.08			0.14	0.05	0.00 (B-C)	1
	C	0.2	0.03	0.02	0.03	0.03	0.04	0.06	0.02	0.03	0.04	0.04	0.03	0.03	0.04	0.05	0.05	0.51 (A-B)	

Leukocyte parameters (Leukocyte count (WBC) and counts within Neutrophil region, Lymphocyte region, Monocyte region, Eosionphil region and Basophil region) in blood samples incubated with two different carbon black nanomaterial concentrations (A – 0.5 mg/mL, B – 1 mg/mL), and control suspension incubated with citrated and phosphate-buffered saline (C). Average value (AVG), standard deviation (STD) and statistical significance of the difference given by the probability (p) are given. Statistically significant differences have also been evaluated for statistical power at α = 0.05 (P).

## Discussion

We considered the effect of CB nanomaterial on biological membranes. We found that CB nanomaterial agglomerated in PBS and in platelet rich plasma (Figure 1). Agglomerates of CB nanomaterial were found to interact with human erythrocytes (Figures 2 and 3). Adhesion of agglomerates did not change the local membrane curvature (Figures 2 and 3). SEM micrographs indicate that the surface of large CB nanomaterial agglomerates in close contact with membrane can be relatively small and discretized (Figure 3D, white arrow). If attached over a large area to a CB nanomaterial agglomerate, erythrocytes may be damaged. Figure 3B shows remnants of a cell attached to huge CB nanomaterial complex. Large holes with bulby circular edge were formed in the membrane of the cell shown in Figure 3B (white arrow) causing the cell interior and exterior to exchange the solutions, and a considerable deformation of the shape. Similarly, a cell shown in Figure 2H was attached to huge CB nanomaterial complex over a large area. However, such events were rarely observed in the SEM micrographs.

Yet the interaction between CB nanomaterial agglomerates and the membrane was strong enough to mediate the bridging interaction between erythrocyte membranes (Figure 2B, white arrow) and induce formation of agglomerate-cell complexes already after an hour of incubation with CB nanomaterial in PBS – diluted blood cell suspensions (Figure 4). Similar structures were formed also in blood plasma (Figure 1D2). Such formations may present mechanical occlusions in the bloodstream.

Incubation of the erythrocyte suspension with CB nanomaterial preserved the average number of intact cells after 24 hours. In the control and in the test sample we observed an increase of the portion of echinocytes with respect to discocytes with time but this process was less prominent in the test sample than in the control sample (Table 1).

In contrast, we observed a deleterious effect of agglomerated CB nanomaterial on GUVs. We found a considerable decrease in the number of GUVs already after 20 minutes of incubation with CB nanomaterial. Although the GUV artificial membrane presents a backbone of biological membranes and therefore shares important similarities with the membrane of an erythrocyte or a more complex cell, the difference between these systems may derive also from the properties of other parts of the experimental system (the composition of the solution, the interactions at the interfaces of contact of the sample and the laboratory material).

When GUVs are formed in the electroformation process, they are connected by a network of nanotubules [27]. This network is torn when GUVs are rinsed out of the electroformation chamber, but its remnants stay attached to the GUVs [28]. Under an optical microscope fresh GUVs appear spherical while the attached nanotubules cannot be observed due to their thinness. If a spontaneous process exists within the observation chamber which removes the lipid molecules from the outer layer, the average mean curvature of the membrane diminishes. To keep the membrane free energy as low as possible the GUV adjusts its shape by integration of the nanotubule(s). Concomitantly, the globular part of the GUV becomes flaccid and its thermal shape fluctuations become visible [28] while the eccentricity of GUVs increases when the protrusions are integrated in the mother vesicle. It is important at which stage of this process the test substance (CB nanomaterial) was added to GUVs. Added CB nanomaterial interacts with solutes, and with surfaces of the observation chamber which affects the osmolarity of the suspension. Water migrates through the phospholipid membrane [29] to equalize its chemical potential. If inserted in hypotonic medium, this mechanism causes GUVs to swell while for large enough differences in chemical potential they burst [30]. Experiments with GUVs were performed immediately after the electroformation when the mother vesicle was almost spherical with hindered possibilities to adjust its shape. As the membrane poorly tolerates stretching, the vesicles which were almost spherical, bursted. This mechanism provides a possible explanation for the decrease of the number of GUVs in the first 20 minutes of observation (Figure 6). Measured 50 minutes after the addition of the CB nanomaterial, the number of GUVs remained more or less unchanged with respect to the measurements at 20 minutes. Also, the remaining GUVs in the test sample exhibited larger average eccentricity than the GUVs in the control sample. In agreement with the proposed mechanism we interpret that the vesicles which have prior to addition of CB nanomaterial integrated enough membrane from the remnants of the nanotubular network were flaccid enough and therefore able to adjust their shape and resist the osmotic shock. Similar osmotic effects took place also in erythrocyte suspension, however, erythrocytes have a relatively low relative volume (in discocytes which prevailed in the samples considered this ratio is below 0.7), so the cells were able to adjust their shape in attaining the osmotic equilibrium. As cells were far from the limiting spherical shapes they did not burst, instead, they adjusted their shape by minimizing the free energy of the membrane with underlying membrane skeleton [31] according to the new conditions. Bursting of GUVs in the experiment (Figure 6) therefore likely reflects the *in vitro* conditions.

Echinocytosis in *in vitro* conditions was observed in the control and in the test sample. The possible mechanisms include interaction of solutes with glass surface of the observation chamber [32], change of the difference between the outer and the inner membrane layer areas due to change in composition or shape of the membrane constituent molecules [33-36], change in the composition of the inner and the outer solution [37] and ATP depletion [38]. CB nanomaterial may interfere with these mechanisms. As the zeta potential is negative, the nanoparticles and their agglomerates attract positively charged molecules in the solution and thereby cause changes of the bulk concentration of solutes, with consequent changes in the Donnan equilibrium including pH and therefrom induced transformations of membrane protein conformation [39]. Also, CB nanomaterial may interact with the glass surfaces of the observation chamber and indirectly affect the glass – induced mechanism responsible for echinocytosis in the control sample. However, the effect of CB nanomaterial in our case suppressed the echincytogenic effect observed in the control sample (Table 1).

We found singular ghosts in the samples treated with CB nanomaterial (Figure 3B) but the membrane of the blood cells did not appear locally deformed due to the presence of CB nanomaterial (Figures 2 and 3). CB nanomaterial also did not cause dramatic changes in platelet shape (Figure 5). The treated and the control samples contained mostly resting platelets while the signs of the platelet activation after 24 hours of incubation with CB nanomaterial (roundness of shape, the presence of the tubular protrusions) were observed also in the control sample and were therefore not ascribed to the effect of CB nanomaterial. Figure 5 for comparison presents also platelets that were incubated with agglomerated ZnO where it can be seen that after 24 hours the platelets were considerably activated, but as previously found [11] this effect can be observed already 3 hours after the addition of ZnO nanoparticles to blood cells.

Further, no differences between CB-treated samples and control samples with respect to number, mass and volume of platelets and shapes of their distributions were found. This indicates lack of evidence for platelet activation which is in agreement with our other results. However, we found statistically significant differences in parameter MPC which measures refractive index of platelets. MPC was suggested as a potential measure of the activation of platelets, however the results are not yet decisive [40]. A change in refractive index of platelets due to incubation with CB nanomaterial (Table 2) could in our experiments be a consequence of adsorption of nanoparticles to the cell surface, (Figure 2).

It was found that the number of leukocytes was statistically significantly increased in samples incubated with CB nanomaterial. Peroxidase reaction staining showed an increase of the number of stained particles that are in blood samples ascribed to eosinophils. We suggest that higher number of leukocytes in CB-treated samples could be a consequence of detecting CB aggregates by the cytometer in the frame within the scatter diagram pertaining to leukocytes and of a decreased adsorption of leukocytes to epruvette walls in samples treated with CB nanomaterial. Higher number of peroxidase stained cells could be due to adsorption of CB nanomaterial to the cell surface, thereby influencing the position of events in the cytometer scatter diagram.

We have incubated blood with CB nanomaterial for 24 hours since this was the interval in which interaction of nanomaterial with membranes took place in our previous experiments with TiO_2_ and ZnO [11]. We used the concentration of nanomaterial of the same order that showed an effect in previous work. In analysis of standard blood parameters we used two different concentrations of CB nanomaterial, however, we observed no difference between samples incubated with different concentrations of nanomaterial (Table 2).

In this work we have focused on the effect of CB nanomaterial on biological membranes as revealed by the change of the erythrocyte, platelet and phospholipid vesicle shape. Activation of platelets can be assessed also by other methods, therefore, further studies should be performed taking into account different aspects of blood coagulation.

Our results do not indicate a CB nanomaterial – induced risk for thromboembolic events due to an increased activation of platelets. However, the risk for occlusion of small vessels is indicated due to the formation of rather large agglomerates which may present mechanical occlusions in the vessels. Adhesion of blood cells to these complexes additionally aggravates the situation. Moreover, by mediating the attractive interaction between membranes, CB nanomaterial accumulated in the erythrocyte dimple can stabilize the shape of the cell that is unfavorable for flow through capillaries. Larger complexes of agglomerated CB nanoparticles present a risk for the occlusion of the vessels even with no cells attached.

The observed effects of interaction of nanomaterial with biological membranes on their biophysical properties present only one viewpoint on the effect of nanomaterial on cells. It was observed that PEGylated gold nanoparticles considerably affected function of erythrocytes (decrease of deformability, oxygen delivering ability, CD47 and ATP content, aggregation of band-3), although they did not cause haemolysis [41]. Moreover, these effects can *in vivo* last for weeks due to biocompatibility of nanoparticles [41]. It was suggested that decreased deformability of erythrocytes could be a consequence of adsorption of gold nanoparticles on the erythrocyte membrane [41]. As we have also observed adsorption of CB (Figure 2) as well as of TiO_2_ and ZnO nanomaterial [11] on the erythrocyte membrane, similar long lasting effects of CB nanomaterial would also be probable *in vivo*. Activation of human platelets was induced by nanodiamonds [42], *in vivo* effect on immune system was induced by uptake of carbon ultrafine particles [43], drug carrier nanoparticles dendrimeres were found to induce procoagulant activity of leukocytes [44] and genotoxic effects of TiO_2_ were reported in leukocytes [45]. On the other hand, carbon nanotubes did not induce acute immune response [46]. To better understand underlying mechanisms, further studies considering different viewpoints and different types of nanomaterial are therefore indicated.

Blood cells are a convenient system for the study of the effect of different substances on cells and biological membranes. Monocytes were found an appropriate system for testing the pyrogeneicity of compounds alternative to rabbit immune response [47-49]. Erythrocytes, platelets and GUVs can also reveal information on the relevant effects. It is however necessary to continue improving the methods for quantification of these effects and expanding the possibilities of the use of blood cells.

Analysis of the sample in the observation chamber involves taking images of the sample at different locations. When comparing the images it can be clearly seen that the sample is not completely homogeneous with respect to the number of cells and cell shapes. Statistical analysis of the representative images of the sample should therefore be performed. We have previously developed a computer aided method to assess an ensemble of representative images and analyzed the effect of different substances on GUVs [50,51]. In this work a statistical approach is used also to compare the concentration and the shape of erythrocytes. We have distinguished between three different types of erythrocyte shapes (discocyte, echinocyte and stomatocyte). As besides the errors of this method, the source of the fluctuations in the average values are also systemic errors in preparing the sample, we presented the results in terms of the portions of cells of a certain type of the shape. A choice of relevant parameters and an iterative approach involving the construction of an ensemble of images representative for the system, calculating the average values and statistical significance of the differences and checking whether the power of the ensemble is large enough will lead to the optimization of the method with respect to the time used for analysis.

The analysis of the potentially prothrombogenic effect of exogenously added substance could be complemented by considering also the extracellular vesicles isolated from blood samples [13,52]. Currently, the most widely used markers for assessment of extracellular vesicles are the antibodies. However, development of ethically superior materials (aptameres) that are produced *in vitro* [53], and the corresponding technologically advanced methods [54] should be considered.

It was reported that various types of nanoparticles and in particular CB, can directly oxidize molecules by surface-mediating reactions and leakage of transition metals and redox-active substances, and thus damage the membranes [3]. CB nanomaterial was also observed to induce apoptosis in HUVECs [55] and in bronchial epithelial cells [56] which was interpreted by leakage of the enzymes due to (mitochondrial) membrane injury [56]. Biochemical approaches also indicate the importance of the integrity, composition and properties of the membrane in vital cell processes and support our indications that understanding basic membrane properties and relevant interactions are crucial in nanotoxicology studies.

Analysis of the shape and population characteristics of erythrocytes and GUVs provides a quantitative method suitable for the study of the effect of added substances on biological membranes and should be considered in the future paradigms of research and testing.

## Conclusions

Carbon black nanomaterial suspended in citrated and phosphate buffered saline interacted with erythrocyte membrane but in 24 hours the direct interactions did not cause observable changes in local membrane shape or in global erythrocyte shape. Also we observed no changes in shape of platelets due to incubation of platelet rich plasma with the nanomaterial within 24 hour observation and in population parameters that consider concentration, volume and distribution shape. The carbon black nanomaterial however formed large agglomerates in sugar solution, in citrated and phosphate buffered saline and in blood plasma. Indirect effects on the artificial membranes were interpreted by changes in osmolarity induced by addition of carbon black nanomaterial to the giant phospholipid vesicle suspensions. Blood cells and giant phospholipid vesicles are appropriate systems for the study of the effects of nanomaterial on biological membranes.

## Methods

### Chemicals

The phospholipid 1-Palmitoyl-2-Oleoyl*-sn-*Glycero-3-Phosphocholine (POPC) and cholesterol were purchased from Avanti Polar Lipids, Inc. (Alabaster, Al, USA). Sucrose and glucose were purchased from Sigma–Aldrich (Steinheim, Germany). Stock solutions (1 mg/mL) of both POPC and cholesterol were prepared by dissolving the lipid in a mixture of CHCl_3_ and MeOH (2:1, v/v). Sucrose, glucose, paraformaldehyde, CHCl_3_ and MeOH were purchased from Sigma–Aldrich (Steinheim, Germany), NaCl, NaH_2_PO_4_⋅2H_2_O, Na_2_HPO_4_⋅2H_2_O and Triton X 100 surfactant (OmniPur) from Merck KGaA (Darmstadt, Germany) and KCl and KH_2_PO_4_ from Kemika (Zagreb, Croatia). Citrated and phosphate buffered saline (PBS): 137 mM NaCl, 2.7 mM KCl, 7.8 mM Na_2_HPO_4_⋅2H_2_O, 1.5 mM KH_2_PO_4_, 10.9 mM Na_3_C_6_H_5_O_7_, pH 7.4) was prepared with ultrapure distilled H_2_O and filtered before use through 0.22 μm pore filters. Glutaraldehyde and osmium tetroxide (OsO_4_) were purchased from SPI Supplies (West Chester, PA USA).

### Carbon black nanomaterial

CB nanopowder was from PlasmaChem GmbH (Berlin, Germany). The average size of primary particles was 13 nm. Stock suspensions of CB nanomaterial were prepared in PBS at 5 mg/ml.

### Characterization and imaging of carbon black nanomaterial suspensions

Characterization of CB nanomaterial (primary characteristics of CB nanomaterial) and nanoparticle suspensions (secondary characteristics of CB nanomaterial) were assessed by SEM and TEM electron microscopy, by DLS and by zeta potential measurements. For TEM, water suspension of nanoparticles was applied on a copper-grid-supported transparent carbon foil at room temperature. TEM imaging of nanomaterial was performed using a JEOL 2100 transmission electron microscope (Tokyo, Japan) operated at 200 kV. SEM imaging of nanomaterial in PBS was performed using LEO Gemini 1530 (LEO, Oberkochen, Germany) scanning electron microscope.

Dispersed CB nanomaterial dissolved in PBS was inspected by DLS using 3D DLS-SLS spectrometer (LS Instruments, Fribourg, Switzerland). Zeta potential of CB nanomaterial in PBS was measured at the pH value of the suspension using ZetaPals (Brookhaven Instruments Corporation, Holtsville, NY, USA).

### Blood sampling

For SEM imaging blood was collected from two authors (female and male, average age 40 years) and a volunteer (female, 30 years) with no record of the disease into 2.7 mL tubes containing 270 μL trisodium citrate at a concentration 0.109 mol/L. Blood was collected by vein puncture with free flow into tubes (BD Vacutainers, Becton Dickinson, CA) by using a 21-gauge needle (length 70 mm, inner radius 0.4 mm) (Microlance, Becton Dickinson, NJ, USA). Sampling was performed according to the Declaration of Helsinki and a written consent to take blood was given by the donors. The study was approved by the National Ethics Committee, No 117/02/10. No adverse effects on donors’ health due to sampling were observed.

For direct observation under the optical microscope, a drop of blood was taken from an author (female, 54 years) with a pipette from a small incision on a fingertip. Blood was inserted into an Eppendorf tube previously filled with 1 mL of PBS and gently mixed by turning the sample upside down.

For the study of population of blood cells, 2.3 mL of blood was collected from 14 volunteers with no record of disease (13 female and 1 male, average age 22 years) into evacuated tubes (BD Vacutainers, Becton Dickinson, CA) containing 270 μL trisodium citrate at a concentration 0.109 mol/L. Blood was collected by vein puncture by using a 21-gauge needle (length 70 mm, inner radius 0.4 mm) (Microlance, Becton Dickinson, NJ, USA). Each subject donated 3 tubes of blood.

### Preparation of blood cells for electron microscopy

Blood was processed within 1 hour from sampling. Blood was centrifuged in a Centric 400R centrifuge (Domel d.o.o., Železniki, Slovenia) at 150 g and 37°C for 10 minutes to separate erythrocytes from platelet rich plasma. Erythrocytes were repeatedly washed with PBS by centrifugation at 1550 g and 37°C for 10 minutes. Washed erythrocytes or platelet rich plasma were aliquoted into equal parts (50 μL in case of erythrocytes and 200 μL in case of platelet rich plasma). CB nanomaterial suspended in PBS (or PBS alone for control) was added to aliquots in v/v ratio of 2:1 in case of erythrocytes and 3:1 in case of platelet rich plasma, 3 and 2 units corresponding to erythrocytes and platelet rich plasma, respectively. After incubation the samples were fixed in 0.1% glutaraldehyde, incubated for another hour at room temperature and centrifuged at 1550 g and 37°C for 10 minutes. Supernatant was exchanged for PBS samples were vortexed, centrifuged at 1550 g and 37°C for 10 minutes and fixed in 2% glutaraldehyde for an hour.

### Phase contrast microscopy of erythrocytes

5 μL of erythrocyte suspension was placed into observation chamber composed of two cover glasses which were glued together with nail polish. Samples were observed under Olympus GWB BH-2 (Olympus Corporation, Tokyo, Japan) microscope with phase contrast optics at objective magnification 400×. Images were taken with Canon EOS 450D digital camera (Canon Inc., Tokyo, Japan).

### Scanning electron microscopy of blood cells

Fixed samples were washed by exchanging supernatant with PBS and incubated for 20 minutes at room temperature. This procedure was repeated 4 times while the last incubation was performed over night at 8°C. Samples were then post-fixed for 60 minutes at 22°C in 1% OsO_4_ dissolved in 0.9% NaCl, dehydrated in a graded series of acetone/water (50%–100%, v/v), critical-point dried, gold-sputtered, and examined using a LEO Gemini 1530 (LEO, Oberkochen, Germany) scanning electron microscope.

### Carbon black nanomaterial effect on erythrocyte shape in populations of erythrocytes

PBS-diluted blood samples incubated with the test or control solution were observed under the Leitz Aristoplan (Leitz, Wetzlar, Germany) optical microscope equipped by the Watec (Model: 902DM3S), Watec Inc., New York, USA, camera and Pinnacle Studio HD, Version 15.0.0.7593 frame graber and software, Avid Technology Inc., USA. Final concentration of CB nanomaterial in samples was 0.5 mg/mL. The samples were observed after 1, 3 and 24 hours, in order to compare the effect with previously considered effect of TiO_2_ and ZnO nanoparticles [11]. The sample was further diluted to obtain a required density of blood cells for observation. The required density rendered a monolayer of relatively closely packed cells in an observation frame under the microscope. An observation chamber 1.5 × 1 cm^2^ was created on the glass by using silicon grease. 40 μL of the test suspension was placed in the observation chamber and closed by the cover glass. Care was taken not to leave voids in the grease boundary in order to prevent evaporation of liquid from the observation chamber. For quantitative analysis of the effect of CB nanomaterial on blood cell membranes we randomly imaged at least 50 frames over the sample. We avoided regions close to the silicon grease. Two independent experiments were performed.

### Image analysis of blood cell populations

For each sample we created an ensemble of pictures from which we omitted pictures of invalid quality. We counted the number of discocytes, echinocytes and stomatocytes in each picture. It was assumed that each picture was representative for the sample and that the number of cells of each type fluctuated around the corresponding average value. The average values of the percent of discocytes, echinocytes and stomatocytes were calculated for each sample. Altogether 10712 cells were included in the final analysis.

### Electroformation of giant unilamellar phospholipid vesicles

The electroformation of GUVs [49] was performed at room temperature. In brief, 40 μL of the lipid mixture of POPC (80%, v/v) and cholesterol (20%, v/v), both dissolved in 2:1 chloroform/methanol mixture, was spread over two platinum electrodes and the solvent was allowed to evaporate in low vacuum for two hours. The electrodes were then placed into the electroformation chamber (2 mL Eppendorf cup), filled with 2 mL of 0.3 M sucrose solution. An alternating electric field was applied as described in [57]. After the electroformation, 600 μL of 0.3 M sucrose solution containing GUVs was added to 1 ml of 0.3 M glucose solution.

### Experiments with giant unilamellar phospholipid vesicles and carbon black nanomaterial

Immediately after the electroformation, the suspension of GUVs in sucrose/glucose solution was mixed by turning the Eppendorf cup upside down (five times). The suspension was diluted (300 μL of 0.3 M glucose solution was added to 100 μl of original vesicle solution) in order to obtain the desired concentration of GUVs, which was found convenient according to our preliminary experiments. Subsequently, the diluted suspension was aliquoted into 2 vials (80 μL in each vial). Two parallels of test suspension of CB nanomaterial (0.05 mg/mL in 0.3 M glucose solution) and two parallels of control suspension (0.3 M glucose solution) were added to GUV suspensions in volume ratio 9:1 (GUVs: test suspension) to reach the final concentration of nanomaterial 10 μg/mL. Vials were turned upside down five times to mix the test suspension with GUV suspension. Thus obtained samples (70 μL each) were separately transferred into four CoverWellTM Perfusion chambers PC4L-0.5 (Grace Bio-Labs Sigma-Aldrich, Steinheim, Germany).

The observation chambers with samples were mounted to the inverted phase contrast light microscope (Nikon Eclipse TE2000-S, Tokyo, Japan). After pre-defined durations of incubation (20 and 50 minutes), GUV populations in each chamber were recorded by a Sony CCD video camera module, model: XC-77 CE (Minato, Japan). These recordings consisted of two video sequences each approximately 2 minutes long. During the recording, the object glass was moved to capture images of thousands of GUVs. With the use of image processing algorithms, the video sequences were transformed into large mosaics [58] where each mosaic contained all vesicles acquired within the sample. GUVs in mosaics were segmented by using a computer-aided approach [58].

A sample of GUVs was incubated with sugar solution for control and another sample was incubated with CB nanomaterial dissolved in sugar solution (0.05 mg/mL). The vesicles were imaged after 20 minutes and after 50 minutes.

The mosaics were separated into images (micrographs) with the size of a single field of view with the microscope at 400× magnification. Using the GUVs’ locations inside the mosaics, all vesicles were associated with the appropriate micrograph allowing us to extract information on vesicle density throughout the micrographs composing the mosaic. Numbers of GUVs in the micrograph were averaged over all micrographs within the test and control samples.

Vesicle shapes were characterized by their deviations from sphere. The nonspherical contours were approximated by fitting with an ellipse with smaller semiaxis *b* and larger semiaxis *a*. The eccentricity ε = (1 - *b*^2^/*a*^2^) ^1/2^ was assigned to each vesicle in the mosaic and the average value for the sample was calculated.

### Analysis of blood cell populations with respect to concentration, volume and distribution

Each subject donated 3 samples of blood to create 3 populations of samples. Population A was incubated with 200 μl PBS-dissolved CB nanomaterial with final concentration 0.5 mg/mL, population B was incubated with equal volume of PBS-dissolved CB nanomaterial with final concentration 1 mg/mL, population C was incubated with equal volume of PBS. Samples were incubated for one hour during which they were continuously slowly turned upside down at room temperature. After one hour the samples were left in upright position for another hour to allow sedimentation of nanomaterial to the bottom of the tubes. Upper milliliter of the samples was pipetted into plastic tubes. After additional 10 minutes, the samples were examined for presence of CB agglomerates. 40 μl of sample was taken with the pipette from the bottom of the tube and placed into an observation chamber created between two cover glasses by silicon grease. No CB agglomerates were observed under the optical microscope Leitz Aristoplan (Leitz, Wetzlar, Germany) equipped by the Watec (Model: 902DM3S), Watec Inc., New York, USA, camera and Pinnacle Studio HD, Version 15.0.0.7593 frame graber and software, Avid Technology Inc., USA. Blood cell populations were assesed by the ADVIA flow cytometer for erythrocyte parameters: erythrocyte concentration RBC, hematocrit HCT, red cell distribution width RDW and hemoglobin distribution width HDW, platelet parameters: concentration of platelets PLT, plateletcrit PCT, mean platelet volume MPV, platelet distribution width PDW, mean platelet complement MPC and mean platelet mass MPM, and leukocyte concentration (WBC).

### Statistical analy**s**is

Methods of descriptive statistics were used to compare the test samples and the respective control samples. We used two-sided pooled *t*-test with equal variance. Probability of the *t*-test below 0.05 was considered statistically significant. The software used for statistical analysis was from R Development Core Team: R: A language and environment for statistical computing [59]. The statistical significance of the *t-* test was given by the probability of the type I or type II error (i.e. by α or β). Power analysis was performed in order to validate the size of samples. Power larger than 0.8 at α = 0.05 indicated the sample of a proper size. For calculation, we used Microsoft Excel software (Microsoft® Office Excel® 2007 SP3) and Power & Sample Size Calculator.

## Abbreviations

CB: Carbon black
GUVs: Giant unilamellar phospholipid vesicles
PBS: Citrated and phosphate-buffered saline
TEM: Transmission electron microscope
SEM: Scanning electron microscope
DLS: Dynamic light scattering
RBC: Concentration of erythrocytes
HCT: Hematocrit
RDW: Red cell distribution width
HDW: Hemoglobin distribution width
PLT: Concentration of platelets
PCT: Plateletcrit
MPV: Mean platelet volume
PDW: Platelet distribution width
MPC: Mean platelet complement
MPM: Mean platelet mass
WBC: Concentration of leukocytes
POPC: 1-Palmitoyl-2-Oleoyl*-sn-*Glycero-3-Phosphocholine
